# Prognostic Biomarkers in Pancreatic Cancer: Avoiding Errata When Using the TCGA Dataset

**DOI:** 10.3390/cancers11010126

**Published:** 2019-01-21

**Authors:** Remy Nicolle, Jerome Raffenne, Valerie Paradis, Anne Couvelard, Aurelien de Reynies, Yuna Blum, Jerome Cros

**Affiliations:** 1 Programme Cartes d’Identité des Tumeurs (CIT), Ligue Nationale Contre le Cancer, 75014 Paris, France; Remy.Nicolle@ligue-cancer.net (R.N.); Aurelien.DeReynies@ligue-cancer.net (A.d.R.); yuna.blum@ligue-cancer.net (Y.B.); 2 INSERM U1149, Beaujon University Hospital, 92110 Clichy, France; raffenne.jerome@gmail.com (J.R.); valerie.paradis@aphp.fr (V.P.); anne.couvelard@aphp.fr (A.C.); 3 Department of Pathology, Beaujon-Bichat University Hospital - Paris Diderot University, 100 Bvd Gal Leclerc, 92110 Clichy, France

**Keywords:** pancreatic cancer, TCGA, curation

## Abstract

Data from the Cancer Genome Atlas (TCGA) are now easily accessible through web-based platforms with tools to assess the prognostic value of molecular alterations. Pancreatic tumors have heterogeneous biology and aggressiveness ranging from the deadly adenocarcinoma (PDAC) to the better prognosis, neuroendocrine tumors. We assessed the availability of the pancreatic cancer TCGA data (TCGA_PAAD) from several repositories and investigated the nature of each sample and how non-PDAC samples impact prognostic biomarker studies. While the clinical and genomic data (*n* = 185) were fairly consistent across all repositories, RNAseq profiles varied from 176 to 185. As a result, 35 RNAseq profiles (18.9%) corresponded to a normal, inflamed pancreas or non-PDAC neoplasms. This information was difficult to obtain. By considering gene expression data as continuous values, the expression of the 5312 and 4221 genes were significantly associated with the progression-free and overall survival respectively. Considering the cohort was not curated, only 4 and 14, respectively, had prognostic value in the PDAC-only cohort. Similarly, mutations in key genes or well-described miRNA lost their prognostic significance in the PDAC-only cohort. Therefore, we propose a web-based application to assess biomarkers in the curated TCGA_PAAD dataset. In conclusion, TCGA_PAAD curation is critical to avoid important biological and clinical biases from non-PDAC samples.

## 1. Introduction

Consortium efforts, such as those of the Cancer Genome Atlas (TCGA) or the International Cancer Genome Consortium (ICGC), to massively sequence thousands of tumors from multiple types have led to a much better understanding of tumor biology. While the data were freely accessible, their use was restricted in practice to teams with great expertise in bioinformatics. Further efforts from centers such as the Broad Institute (https://gdac.broadinstitute.org) or the University of California Santa Cruz (http://xena.ucsc.edu) allowed easy access to TCGA normalized RNAseq, methylation and clinical data, often readily available in excel files. Finally, multiple web-based platforms were launched with “one-click” capabilities to give users direct access to the prognostic role of the gene expression level, the frequency of any mutation, the protein level expression and the networks of genes and proteins, etc. Main platforms include TCGA (https://gdc.cancer.gov), the Broad Institute (https://gdac.broadinstitute.org), the University of California Santa Cruz (http://xena.ucsc.edu), cBioportal (http://www.cbioportal.org) and the Human Protein Atlas (https://www.proteinatlas.org). These platforms have given everyone access to use these data as exploratory or validation sets.

Pancreatic cancer is a generic term often misused as a surrogate for the most common malignant tumor entity in this organ, the ductal adenocarcinoma (PDAC). Other malignant tumor entities such as the neuroendocrine neoplasms or the acinar cell carcinomas can also be found in the pancreas [1]. While these tumors are uncommon, they often fall under the umbrella term of “pancreatic cancer”, and are as such defined by completely different biology (mutational and transcriptional profiles) and clinical outcomes from the classical PDAC [2,3]. If such tumors were unnoticeably included within a dataset mainly composed of PDAC, they may introduce a strong bias in data analysis and lead to false conclusions regarding the prognostic value of a DNA mutation or an mRNA expression level. Depending on the source, 176 to 185 samples compose the TCGA study dedicated to the pancreas (TCGA_PAAD) and the multiomic analysis of these samples from the TCGA group was restricted to 150 samples [4]. In a recent study, Peran et al. highlighted that the TCGA_PAAD cohort, which mostly comprised patients who underwent surgery, displayed a much better survival rate than that of the unselected cohort SEER [5]. They further demonstrated that a failure to exclude non-PDAC samples might introduce a bias in the gene expression analyses leading to false conclusions being drawn when assessing the prognostic value of several mRNAs.

In this study, we aim to gather and compare the available data concerning the TCGA_PAAD from all the main repositories and clearly establish a list of suitable TCGA_PAAD samples for the PDAC centered studies. We then compare the TCGA_PAAD cohort to a large multicentric consecutive cohort of surgical PDAC. Using the key DNA mutation and the whole transcriptome, we then assessed the potential for bias based on survival analyses when using an uncurated sample list. Finally, we designed a web-based tool to assess the prognostic value of any gene expression on the curated TCGA_PAAD dataset.

## 2. Results

### 2.1. Data Comparison from the Repositories for the TCGA_PAAD

Across most platforms queried, the number of patients within the TCGA_PAAD study was consistent and set at 185 (TCGA data portal *n* = 185, UCSC Xena *n* = 185, Broad Institute Firehose *n* = 185, The Human Protein Atlas *n* = 176 (only patients with available RNAseq data were considered) and cBioportal *n* = 185). In all platforms, clinical data were available for the 185 patients. Depending on the platforms, mutation and copy number data were available for 184 or 185 samples, DNA methylation data were consistent and available for 184 samples, and RNAseq data were the most discordant, ranging from 176 to 185 samples (cBioportal *n* = 185, UCS Xena *n* = 183, Broad Institute Firehose *n* = 178, TCGA data portal *n* = 178 and The Human Protein Atlas *n* = 176). TCGA-derived RNAseq data were the most frequently used. As a result, we carefully investigated the nature of the samples to explain the discrepancy in the available number of samples depending on the platforms. RNAseq data were not available for seven patients and the number of patients with RNAseq data (list in Table S1) was set at 178. For four patients, RNAseq data from the normal adjacent pancreas were available and included in the datasets from the platforms with more than 183 samples. These samples could be identified, as they have the same ID number as the tumor sample but with “-11” at the end of their ID instead of “-01” (TCGA-HV-A5A3-01 and TCGA-HV-A5A3-11, for instance). For one patient, RNAseq data from the primary tumor and the metastasis was available. The metastasis is identifiable by its “-06” at the end of the sample ID TCGA-HZ-A9TJ-01 (primary tumor) and TCGA-HZ-A9TJ-06 (metastasis). This highlighted that data retrieval must be done with care, and confirms that while genomic and clinical data are available for 185 patients, RNAseq data are only available for 178 unique patients.

### 2.2. Curation of the TCGA_PAAD Dataset

**Figure 1:**
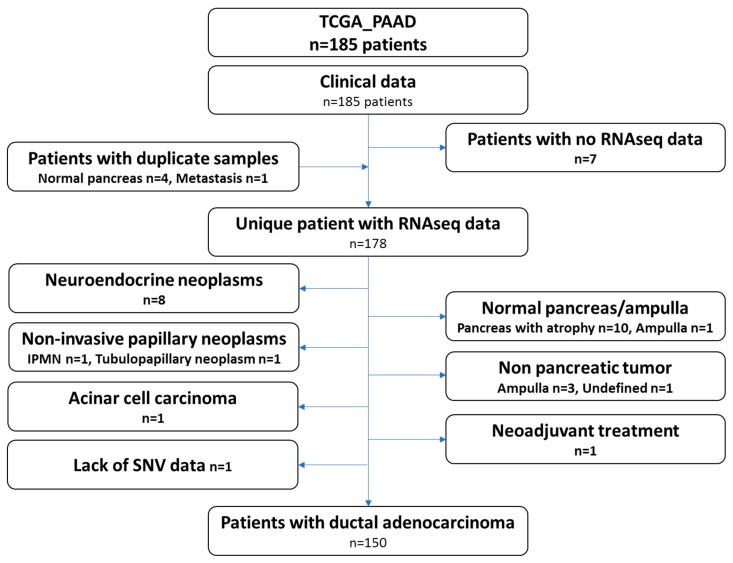
Flow chart depicting the curation of the pancreatic cancer dataset (TCGA_PAAD).

The recent study from the TCGA group focused on pancreatic ductal adenocarcinoma and only included 150 samples. Therefore, we carefully reviewed the clinical and histological data of all the cases gathered through the repositories and by viewing the virtual slides. Ten samples presented the pancreas as normal with atrophy, eight samples were neuroendocrine neoplasms, four samples were tumors derived from other organs (duodenum-ampulla in three cases and undefined location in one case), two samples were intraductal papillary neoplasms, one sample was an acinar cell carcinoma, one was a ductal adenocarcinoma but had received neoadjuvant chemotherapy and one had a normal ampulla. It should be noted that for several patients, while the analyzed specimens were not PDAC (atrophic pancreas, PanIN, etc.), these patients did have a PDAC. These clinical data may therefore be used, but not the omic data. One additional case was excluded, as no single nucleotide variation data was available (TCGA-L1-A7W4-01). This sample was listed as a PDAC and treated with adjuvant gemcitabine, a classical drug for this tumor. The examination of the frozen section showed a poorly differentiated tumor, which was difficult to clearly identify as a PDAC or a neuroendocrine carcinoma. Copy number abnormalities (*SMAD4* deletion, *MYC* amplification, no alteration on *TP53*, *CDKN2A* and *RB1*) did not help in further assuring the diagnosis. The flow chart presenting the TCGA-PAAD sample curation is presented in Figure 1 and the full list of the 150 proper PDAC sample and the 28 non PDAC sample are provided in Table S1.

### 2.3. Clinical Relevance of the TCGA_PAAD Curated Sample List

**Figure 2:**
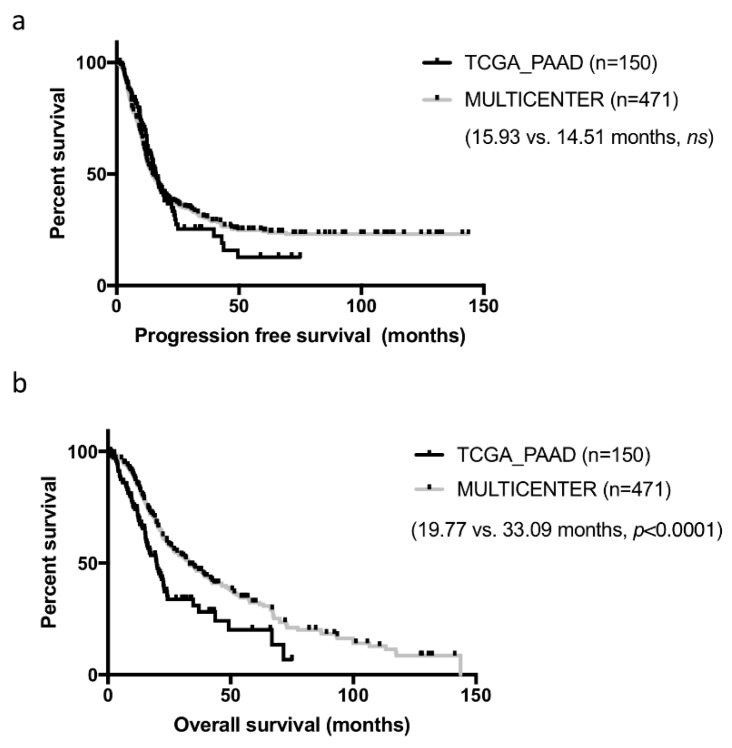
Progression-free and overall survival of the curated TCGA_PAAD and a PDAC multicenter cohort. Kaplan-Meier curves depicting the progression-free (**a**) and overall survival (**b**) of the curated TCGA-PAAD cohort (*n* = 150) and a multicenter PDAC cohort (*n* = 471).

Samples constituting of the TCGA cohorts were collected from multiple institutions, which may have introduced some heterogeneity in patient management and clinical data collection. In addition, while these cases were all surgical resections, they were not consecutive. To assess how representative the curated TCGA_PAAD cohort was, we compared it to our well characterized cohort of 471 consecutive resected PDAC in five centers collected over a 13 years period [6]. Basic clinical and pathological comparison is presented in Table 1. For this comparison, we used the consensual 150 cases, but the seven PDAC cases with no RNAseq data could have been added (results unchanged, data not shown). Both cohorts were comparable on most criteria. Tumors from the TCGA_PAAD study tended to be slightly more aggressive with larger and more poorly differentiated tumors (*p* < 0.05). Progression-free survival (PFS) was comparable in both cohorts (TCGA_PAAD 16.75 months vs. 14.51 months in our cohort, *ns*) (Figure 2a). In contrast, overall survival (OS) was much shorter in the TCGA_PAAD cohort (19.54 vs. 33.09 months, *p* < 0.0001) (Figure 2b).

### 2.4. Bias in the Prognostic Value when Using the Uncurated Cohort

**Figure 3:**
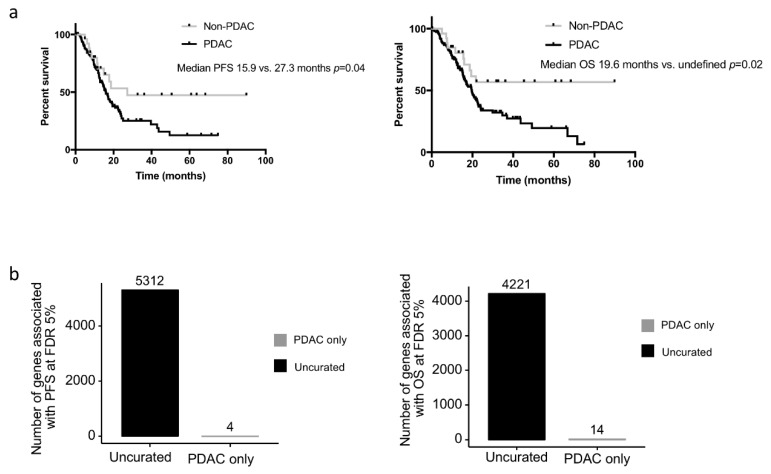
Progression-free and overall survival of the PDAC and non-PDAC cases. (**a**) Kaplan-Meier curves depicting the progression-free (left panel) and overall survival (right panel) of the PDAC cases (*n* = 150) and the non-PDAC cases (*n* = 27). (**b**) Number of genes associated significantly associated with the progression-free (left panel) and overall survival (right panel) in PDAC only cases and the uncurated cohort.

**Figure 4:**
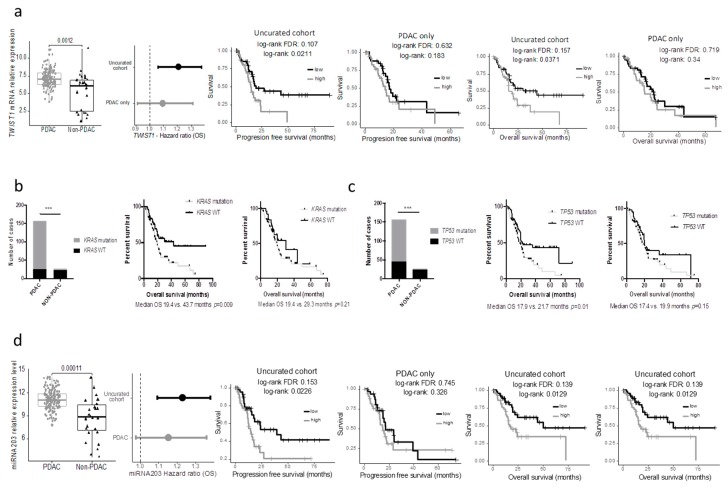
Bias in prognostic analysis when using the uncurated cohort. (**a**) *TWIST1* mRNA expression in PDAC and non-PDAC cases and prognostic impact (OS) in the uncurated and the PDAC only cohorts (left panels). Kaplan-Meier curves depicting the overall (middle panel) and progression-free survival (right panels) according to *TWIST1* expression in the uncurated cohort or the PDAC only cohort. (**b**) and (**c**) Distribution of the *KRAS* and *TP53* mutation in the PDAC and non-PDAC cases (left panels) and Kaplan-Meier curves depicting the overall survival according to the mutational status in the uncurated cohort (middle panels) or the PDAC-only cohort (right panels). (**d**) miR-203 mRNA expression in PDAC and non-PDAC cases and prognostic impact (OS) in the uncurated and the PDAC only cohorts (left panels). Kaplan-Meier curves depicting the overall (middle panel) and progression-free survival (right panels) according to miR-203 expression in the uncurated cohort or the PDAC only cohort.

We assessed whether the clinical or biological data from the non-PDAC patients impacted survival analyses performed on the TCGA_PAAD cohort. While the median PFS and OS of the uncurated cohort was marginally longer than that of the pure PDAC cohort (median PFS: 17.0 versus. 15.9 months and median OS 20.1 vs. 20.2 months respectively), the non-PDAC patients had a much longer PFS and OS compared to the pure PDAC cohort (*n* = 150) (median PFS: 27.3 vs. 15.9 months *p* = 0.07; median OS: unattained vs. 19.6 months *p* = 0.03; five year overall survival: 54.6% vs. 19.7%) (Figure 3a.). When the gene expression data were considered as continuous values; 5312 and 4221 genes were significantly associated (FDR 5%) with the PFS and OS respectively in the uncurated cohort (where a total of 17,302 genes were tested, uncorrected log-rank test: 6618 and 7260 genes at alpha = 5%). In contrast, if the PDAC-only cohort was considered, only 4 and 14 genes were significantly associated with the PFS and OS respectively (at FDR 5%; uncorrected log-rank test: 2632 and 2374 genes at alpha = 5%) (Figure 3b). In the PDAC-only cohort, 2671 genes were significantly over expressed and 1730 genes were significantly under expressed compared to the non-PDAC samples of the cohort (Table S2). Using a median cut off, 594 and 409 genes were associated with progression-free and overall survival, respectively, in the whole cohort (at FDR 5%; uncorrected log-rank test: 3907 and 3776 genes at alpha = 5%), while only 3 and 0 in the PDAC-only subgroup (at FDR 5%; uncorrected log-rank test: 1706 and 2062 genes at alpha = 5%). Within the genes that were differentially expressed in the 2 cohorts, we handpicked genes that were previously described as having a strong impact on prognosis. The progression-free and overall survival of cases with the top and bottom 25% expression were then compared in the uncurated and the curated cohort. While some genes such as *ERBB2*, *HK2, SLC2A1* were significantly or nearly significantly associated with the prognosis in both cohorts, others such as *MUC1/LOX/TWIST1/PI3K* lost their prognostic significance in the pure PDAC cohort (*TWIST1* as an example in Figure 4a).

Similar findings were observed when key mutations in PDAC were assessed. Here we included the seven PDAC cases with the DNA data available (but no RNAseq). The results were similar when using the minimal 150 PDAC cohort. While the mutational status of *KRAS* and *TP53* were strongly associated with the overall survival in the uncurated cohort, these mutations lost their prognostic significance in the pure PDAC cohort (Figure 3b,c). In a recent study, Shi et al. searched in silico miRNAs associated with the prognostic significance in the TCGA_PAAD cohort and reported that a five miRNA signature had a strong prognostic value [7]. Unfortunately, they used the uncurated cohort. As a consequence, when these miRNAs were assessed in the pure PDAC cohort, they lost their prognostic significance (mir-203 as an example, Figure 4d).

### 2.5. Web-Based Application to Query the Curated TCGA-PAAD Dataset

In order to quickly assess the prognostic significance of a gene in the curated TCGA_PPAD, we developed a web-based application [8]. The application requires a gene symbol and displays survival curves by splitting patients into groups depending on their level of expression of the selected gene, if it is available in the TCGA_PAAD RNAseq data. The patients are either separated in two groups, low versus high, or separated by a given percentile cut, 50% by default. In this splitting case, all 144 patients were used for the survival analysis. The patients could have also been divided by *interval* in which only the patients with the highest expression levels would be shown against the patients with the lowest levels of expression of the analyzed gene. By default, the upper quartile (>75%) was shown against the lower quartile (<25%). In both types of survival analysis, the *p*-value of a log-rank test was shown, as well as the median survival in each group. The log-rank test *p*-value and the Hazard Ratio of the continuous gene expression level were also shown.

## 3. Discussion

Clinical validation of their findings is often a bottleneck step for basic scientific laboratories. Consortium efforts such as those of the TCGA and the ICGC have been a tremendous help for this purpose. In addition, it facilitated alternative approaches based on in silico discovery completed by clinical validation. Here, we presented a thorough analysis of the TCGA dataset, which was dedicated to pancreatic neoplasms and highlighted the heterogeneity of the data sources and samples. In addition, we demonstrated that the curation of this dataset led to the exclusion of almost 20% of the cases but was a mandatory step, as it prevented false results on prognostic analyses.

TCGA data may be retrieved through numerous platforms. Confusion for researchers may arise from the fact that while data for a particular sample are homogeneous across all platforms, the list of available samples is heterogeneous with little to no information on the sample of most platforms. In addition, while the genomic data for a sample may be exploitable, the RNAseq data may not. The TCGA data portal provided the most comprehensive information on the nature of the samples, but it required a deeper exploration within the platform to find it. Due to most platforms providing the official clinical data, but not a detailed manifest of the samples analyzed, it is important to retrieve the final list of “good samples” and to only assess these, a function available on some platforms but not all.

In the TCGA_PAAD, there were three main reasons for sample exclusion. The first reason, valid across all the data (DNA, RNA, etc.) was the histology of the tumors. PDAC have a well-described biology (mutational and transcriptional patterns) and a clinical behavior, which is very distinct from other pancreatic neoplasm such as neuroendocrine neoplasms (NEN), acinar cell carcinomas or intraductal papillary neoplasm (IPMN). Well-differentiated NEN for instance, have a completely different mutational and transcriptional pattern from PDAC and a much longer survival rate. Therefore, any alteration specific to PDAC will artificially see its prognostic value increased. The second reason was that the sample analyzed was not a tumor. It was either a normal pancreas or from a PDAC-look alike histological lesion, often atrophic fibrosis with a stroma-like appearance. These patients had a prolonged survival compared to PDAC patients. As a result, the prognostic value was strongly biased in any alteration present in PDAC. Finally, for one sample, the single nucleotide variation data were not available (TCGA-L1-A7W4-01).

Peran et al. compared the clinical characteristics of the TCGA_PAAD cohort with that of the SEER and the national cancer database and reported that the TCGA_PAAD cohort had less locally advanced and metastatic tumors and therefore a much better chance of survival [5]. This highlights another potential bias in the cohorts required for this type of multi-omics analyses. As they require frozen tumor material, the cohorts usually include only surgical specimens. This is an important bias for PDAC as only 15% of patients present with a resectable tumor. In addition, only large tumors have frozen material set apart usually, leading to a nonconsecutive series. Yet, when comparing the pure-PDAC TCGA cohort with our large multicentric consecutive cohort, we have found few differences, except for the median tumor size and therefore a slightly worse overall survival. This confirmed the clinical validity of the curated cohort for prognostic studies.

The importance of the curation is also highlighted by the massive prognostic bias introduced by the samples (tumor or non-tumor) with a different molecular profile from the PDAC and a prolonged survival. We observed that many molecular alterations (gene mutation, mRNA or miRNA aberrant expression level) had a prognostic value only in the uncurated cohort. This is not surprising as most of these were absent from the good prognostic non-PDAC subgroup. Improper data curation led to the description of many PDAC prognostic factors that lost their value in the curated dataset [9,10]. This is in line with the study of Peran et al. that described on a limited number of genes how the use of a curated cohort led to the loss of their prognostic value.

We therefore developed a free web-based app to quickly assess the prognostic value (progression-free and overall survival) of the expression of any gene on the curated dataset. This is to our knowledge the only “click and play” tool to reliably assess the prognostic value of any gene using either a cut off based on the median expression value or any interval, and retrieve the survival and expression data.

## 4. Patients and Methods

### 4.1. Data Query from the Main Data Repository

The following data repository were queried to retrieve a sample list and biological annotations, whole normalized level 3 RNAseq and miRNA data, specific DNA mutation data (*KRAS*, *TP53*, *SMAD4*) and clinical data: TCGA (https://gdc.cancer.gov), the Broad Institute (https://gdac.broadinstitute.org), the University of California Santa Cruz (http://xena.ucsc.edu), cBioportal (http://www.cbioportal.org) and the Human Protein Atlas (https://www.proteinatlas.org). The sample list and the clinical data used in the TCGA group publication were also retrieved from the supplemental data [5].

### 4.2. Data from the Multicenter Ductal Adenocarcinoma Cohort

For comparison with the TCGA_PAAD cohort, we used a previously published cohort of 471 consecutive patients who underwent curative intent surgery for PDAC at 5 university centers between September 1996 and August 2009. Subjects were excluded if they had received preoperative treatment and macroscopically incomplete resection (R2) or if their tumor histology was not a ductal adenocarcinoma. Patients who died of postoperative complications within 30 days following surgery were also excluded.

### 4.3. Data Analysis

Survival analyses with gene expression were performed in R using the *survival* package. The gene expression or miRNA expression association to survival was evaluated by fitting a Cox proportional hazards regression model. The explanatory variable of the survival model fitted for each gene was either the continuous expression values or the discretized expression value (e.g., below versus over the median expression). The association to survival in the curated or uncurated cohorts was assessed for 17,302 genes. For each of them, the p-value of the log-rank test was retrieved and corrected using the Benjamini-Hochberg multiple comparison approach for controlling the false discovery rate. When defining the number of prognostic genes, the log-rank test was used and adjusted to obtain a False Discovery Rate of 5%.

Clinical characteristics of the two cohorts were compared using a Chi2-based test of equal proportions for discrete variables and a Student’s t-test for continuous variables.

Survival curves were drawn using the *ggsurv* function from the *GGally* R package.

### 4.4. Web Based App

A web app to associate gene expression to overall and progression-free survival in the 144 TCGA patients (6 of the 150 curated sample/patient pair had missing clinical data for effective PFS analysis) is available at [8] (executable code available: [11]).

The TCGA data used for the application were obtained from the Broad’s Institute firehose portal (https://gdac.broadinstitute.org, 20,160,128 release). The PDAC survival web application can be used to associate gene expression to overall and progression-free survival in three different ways: by splitting the series of patients into two subseries around a gene expression threshold (e.g., median), by separating the series of patients into two extremes intervals (e.g., upper quartile vs. lower quartile) or by directly associating the continuous gene expression values to the survival. For the splitting and interval analysis, the thresholds can be modified by the user which will update the results accordingly. Kaplan-Meier curves are used and the log-rank test p-values are shown. In addition, the raw survival and gene expression values are shown in order to let anyone reuse the curated data with any software.

## 5. Conclusions

In conclusion, we highlighted in this study the heterogeneity of the data available through the main repositories and the lack of a proper sample description leading to the inclusion in many studies dedicated to PDAC of non-PDAC tumor samples or even non-tumor samples. We confirmed that it introduced a major bias in biomarker prognostic value analysis and we provided a comprehensive list of the curated dataset together with a free web-based app to assess on the curated dataset the prognostic value of gene expression levels.

## Figures and Tables

**Table 1:** Clinical comparison of the TCGA_PAAD and the pancreatic adenocarcinoma multicenter cohort.

Clinical/Pathological Features	TCGA_PAAD (*n* = 150)	PDAC Multicenter Cohort (*n* = 471)	*p*-Value
**Age at diagnostic** (avg. (min, max))	64.89 (35, 88)	63.31 (34, 88)	0.12
**Sex** (male proportion (female, male))	54% (69, 81)	54% (215, 256)	1
**Tumor size** (avg. (min, max))	37.97 (18, 120)	32.42 (7, 150)	1.86 × 10^−4^
**Tumor grade**			< 1 × 10^−10^
G1	5	201	< 1 × 10^−10^
G2	75	189	0.079
G3	69	67	< 1 × 10^−10^
G4	1	0	0.5579
**Pathology TNM**			0.0047
T1	1	17	0.114
T2	20	68	0.861
T3	125	386	0.676
T4	3	0	0.016
N (N1 proportion (N0, N1))	73.8% (39, 110)	74.5% (120, 351)	0.950
M (M0 proportion (M0, M1))	94.4% (68, 0)	100% (471, 0)	1.11 × 10^−5^

## Supplementary Materials

The following are available online at http://www.mdpi.com/2072-6694/11/1/126/s1, Table S1: TCGA_PAAD sample list and biological nature of the samples, Table S2: Comparison of genes associated with PFS and OS in the uncurated and the curated TCGA_PAAD cohorts.
